# Advantage of Using Allele-Specific Copy Numbers When Testing for Association in Regions with Common Copy Number Variants

**DOI:** 10.1371/journal.pone.0075350

**Published:** 2013-09-10

**Authors:** Gaëlle Marenne, Stephen J. Chanock, Núria Malats, Emmanuelle Génin

**Affiliations:** 1 Inserm UMR-S946, Univ. Paris Diderot, Institut Universitaire d’Hématologie, Paris, France; 2 Genetic and Molecular Epidemiology Group, Spanish National Cancer Research Centre (CNIO), Madrid, Spain; 3 Division of Cancer Epidemiology and Genetics, National Cancer Institute, Department of Health and Human Services, Bethesda, Maryland, United States of America; 4 Inserm UMR-1078, Univ. Bretagne Occidentale, Brest, France; 5 Centre Hospitalier Régional Universitaire de Brest, Brest, France; Cleveland Clinic Lerner Research Institute, United States of America

## Abstract

Copy number variants (CNV) can be called from SNP-arrays; however, few studies have attempted to combine both CNV and SNP calls to test for association with complex diseases. Even when SNPs are located within CNVs, two separate association analyses are necessary, to compare the distribution of bi-allelic genotypes in cases and controls (referred to as SNP-only strategy) and the number of copies of a region (referred to as CNV-only strategy). However, when disease susceptibility is actually associated with allele specific copy-number states, the two strategies may not yield comparable results, raising a series of questions about the optimal analytical approach. We performed simulations of the performance of association testing under different scenarios that varied genotype frequencies and inheritance models. We show that the SNP-only strategy lacks power under most scenarios when the SNP is located within a CNV; frequently it is excluded from analysis as it does not pass quality control metrics either because of an increased rate of missing calls or a departure from fitness for Hardy-Weinberg proportion. The CNV-only strategy also lacks power because the association testing depends on the allele which copy number varies. The combined strategy performs well in most of the scenarios. Hence, we advocate the use of this combined strategy when testing for association with SNPs located within CNVs.

## Introduction

Different types of variations occur in the human genome, ranging from single nucleotide base changes to copy changes of entire chromosomes (e.g., trisomies). Single nucleotide polymorphisms (SNPs) are single base changes of the DNA sequence that cover less than 1% of the human genome. Structural variations are larger polymorphisms that involve contiguous sequences of nucleotides [1]. Among them, copy number variants (CNVs) are defined as genomic regions larger than 1kb and present in a variable number of copies in a population [2]. CNVs are distributed throughout the genome and previous works reported that they cover between 3.7% and 12% of the human genome [3,4].

Many SNPs have been shown to contribute to a range of complex diseases involving both genetic and environmental factors through complex mechanisms. Although the role of common CNVs in human diseases has been less investigated, their contribution to complex diseases is probably substantial. Indeed, CNVs often include important functional elements of the DNA such as genes [3,4]. In addition, correlations between CNVs and gene expression have been reported [5]. Furthermore, some CNVs have been found associated with diseases, mainly with neuropsychiatric disorders [6,7].

The recent developments of SNP genotyping technologies have resulted in the large scale analysis of genotypes in genome-wide associations (GWAS), which have identified over 1200 new loci associated with human diseases or traits [8,9]. Data from SNP-arrays can also be used to characterize CNVs using specifically-developed CNV detection algorithms [10–12]. CNV detection from SNP-arrays lacks accuracy [13,14] but presents the advantage of giving information on both the SNP allele and the number of copies.

Given that CNVs are distributed across the human genome, a significant proportion of the SNPs are located in CNVs. While it is biologically plausible that both number of copies and the actual alleles could play a role in disease susceptibility [11,15], very few analyses so far have taken them into account to test their effect simultaneously [16,17].

The objective of this work is to assess the performances of classically used statistical models to estimate the effect of the allele or the number of copies for a disease susceptibility SNP, when it is located within a CNV. The performances of various strategies to simultaneously test for both effects are investigated by simulations of allele-specific copy number states (A, AA, AB, ABB…) in different scenarios of genotype frequencies and inheritance models and compared on real data from HapMap.

## Results

**Figure 1:**
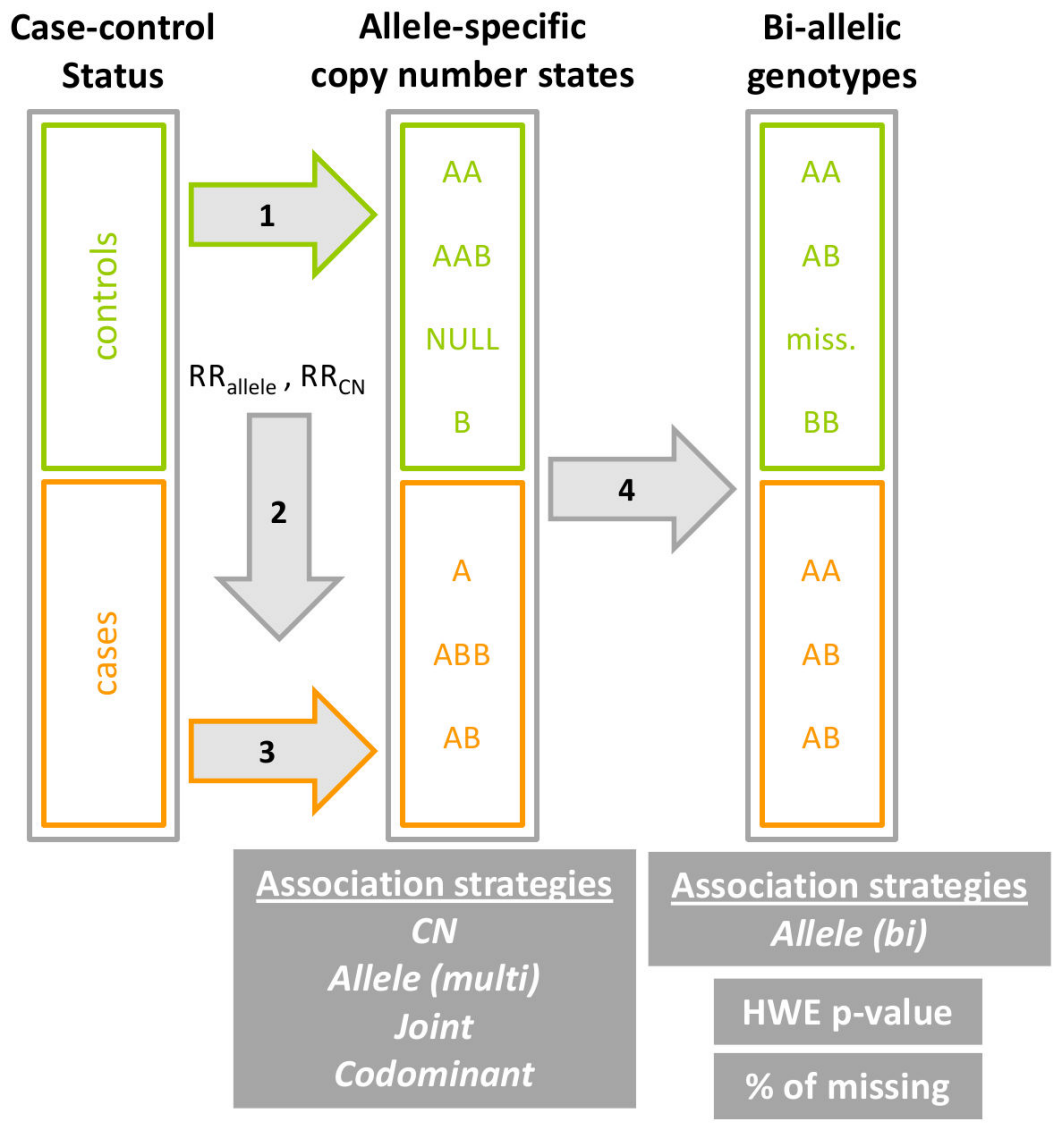
Flowchart of the simulations. 1) The allele-specific copy number states were simulated for controls given the expected frequencies. 2) The expected frequencies in the case population were calculated given the frequencies in the control population and the relative risks (RR) of both the allelic and the copy number effects (RR_allele_ and RR_CN_ respectively). 3) The allele-specific copy-number states were simulated for cases given the expected frequencies in cases. 4) We deducted the bi-allelic genotypes information from the allele-specific copy-number states, given the probabilities of each allele-specific copy-number state to be respectively called as AA, AB, BB or missing. Association tests were performed on both the allele-specific copy-number states that contain the complete information on allele and copy-number, and the bi-allelic genotypes that contain partial information on the allele. Classical criteria used in SNP analysis such as the Hardy-Weinberg equilibrium (HWE) departure in controls and the percentage (%) of missing data were computed on the bi-allelic genotypes.

We modeled disease susceptibility across a genomic region in which deletions and duplications occur independently with respective probabilities f(del) and f(dup) to give rise to 5 possible copy-number states (from 0 copy ,CN=0, to 4 copies, CN=4). In each genomic region, we assumed there was a SNP with two alleles A and B with allele B conferring an increased risk for the disease under study. We assumed that the effects of the B allele and the copy-numbers were multiplicative and we let RR_allele_ and RR_CN_ be respectively the relative risks for the disease associated to one extra B allele and one extra copy number. Using this model, we simulated allele-specific copy number states in 1,000 cases and 1,000 controls, as well as in 5,000 cases and 5,000 controls to see how results were affected by the sample size. These allele-specific copy number states were either analyzed directly or after condensing to bi-allelic genotypes (AA, AB, BB or missing) (Figure 1). Five association strategies were compared testing either for a trend effect of the number of B alleles (referred to as *Allele* (*multi*) or *Allele* (*bi*) strategies), a trend effect of the number of copies (the *CN* strategy), or both allelic and copy number effects (the *Joint* and *Codominant* strategies) (Table 1). The strategies used either the allele-specific copy-number state information (the *Allele* (*multi*), *CN*, *Joint* and *Codominant* strategies), or the bi-allelic genotype information inferred from it (the *Allele* (*bi*) strategy) (see the Material and Methods section for more details). All the strategies used a likelihood-ratio test (LRT) and their null hypotheses are shown in Table 1.

**Table 1:** Details on the different statistical strategies.

**Strategies**	**Dataset used**	**Tested effect**	**Logistic regression model**	**Null hypothesis**
*CN*	allele-specific copy-number	copy-number	logit(p) ~ α + β (A+B)	OR_CN_=1
*Allele (bi*)	bi-allelic genotypes	allele	logit(p) ~ α + β (B)	OR_allele_= 1
*Allele (multi*)	allele-specific copy-number	allele	logit(p) ~ α + β (B*)	OR_allele_= 1
*Joint*	allele-specific copy-number	allele and copy-number	logit(p) ~ α + β_1_(A+B) + β_2_ (A-B)	OR_allele|CN_= OR_CN|allele_=1
*Codominant*	allele-specific copy-number	each allele-specific copy-number genotypes	logit(p) ~ α + Σ β_i_ state_i_	OR_G_=1 for all state G taking AA as reference

A and B refer to the number of alleles A and alleles B respectively in the allele-specific copy-number state. B* refer to the number of alleles B in the bi-allelic genotype. The states used in the logistic regression model for the *Codominant* strategy are the observed allele-specific copy-number states. The null hypotheses were tested by applying likelihood ratio tests.

First, we focused on the situation in which the full information on allele-specific copy number states was available for both cases and controls. This implied that these states were reconstructed from the observed Log R ratio and B allele frequencies using a CNV calling algorithm. For the current model, we assumed here that these reconstructions were unambiguous but we recognize that this is not generally the case on real data as discussed below.

When case-control data were simulated under the null hypothesis of no effect of the allele or of the number of copies (RR_allele_=1 and RR_CN_=1), under most of the scenarios investigated, type-one error rates were equal to their nominal values (Figure S1). The only exception was observed for the codominant model when testing for the effect of each allele-specific copy number state (*Codominant* strategy) with a low frequency of duplications. This could be due to the high number of degrees of freedom and the low number of observations for some allele-specific copy number state categories, but the inflation was small (the maximum type-1-error was 7.4% instead of 5%).

**Figure 2:**
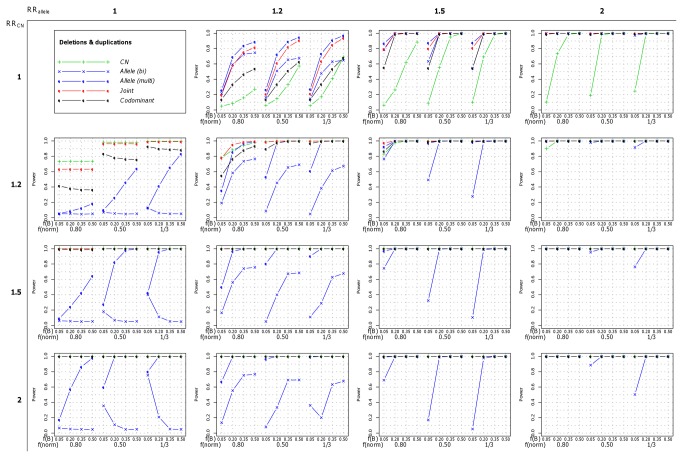
Power of each of the five investigated strategies when both deletions and duplications were present. The power was computed for a sample of 1,000 cases and 1,000 controls under the different scenarios of risk and frequencies. On the X axis, f(B) and f(norm) refer respectively to the frequency of the allele B and to the frequency of normal chromosome carrying one copy of the CNV.

**Figure 3:**
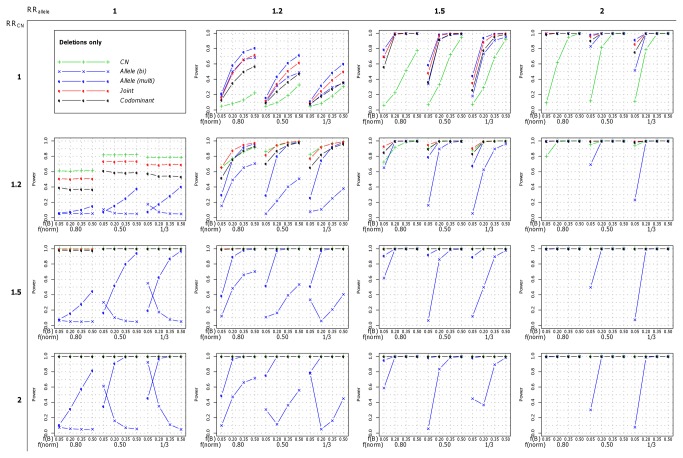
Power of each of the five investigated strategies when only deletions were present. The power was computed for a sample of 1,000 cases and 1,000 controls under the different scenarios of risk and frequencies. On the X axis, f(B) and f(norm) refer respectively to the frequency of the allele B and to the frequency of normal chromosome carrying one copy of the CNV.

**Figure 4:**
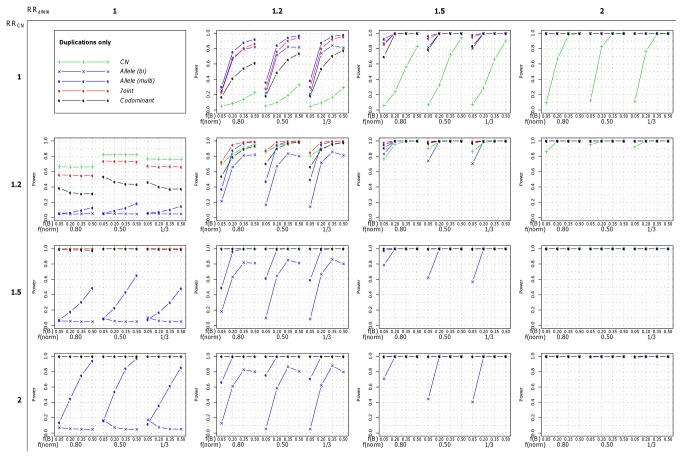
Power of each of the five investigated strategies when only duplications were present. The power was computed for a sample of 1,000 cases and 1,000 controls under the different scenarios of risk and frequencies. On the X axis, f(B) and f(norm) refer respectively to the frequency of the allele B and to the frequency of normal chromosome carrying one copy of the CNV.

We then simulated a single effect of either the allele or the number of copies (RR_allele_>1 and RR_CN_=1, or RR_allele_=1 and RR_CN_>1, respectively). In these situations the most powerful strategies to detect the association were, respectively, the *Allele* (*multi*) and *CN* strategies that were also the ones best fitting the simulated model of association. In the dataset of 1,000 cases and 1,000 controls, the power was maximum (100%) for the relative risks of 1.5 and above. This was true when both deletions and duplications were present (Figure 2, first column for CN effect only and first line for allelic effect only), but also when only one type of copy-number variation was present (Figure 3 for deletions and Figure 4 for duplications). In the dataset of 5,000 cases and 5,000 controls, the power was maximum for relative risks greater than 1.2 (data not show). Interestingly, under these scenarios where a single effect was simulated, the two strategies testing for both effects (the *Joint* and the *Codominant* strategies) performed well and the loss in power compared to the best strategy was relatively low. This was especially true for the *Joint* strategy relative power in comparison to the most powerful strategy (*CN* strategy when RR_allele_=1 and RR_CN_>1 and *Allele* (*multi*) strategy when RR_allele_>1 and RR_CN_=1) was always greater than 0.76. Its relative power even reached 1 when the simulated relative risks, RR_CN_ and RR_allele_ respectively, were greater than 1.5 (Table S1). As expected, the strategies that test for the other effect (i.e the *Allele* (*multi*) strategy when copy-number effects were simulated and the *CN* strategy when B allele effects were simulated) were the less powerful but interestingly the power was not null, thus detecting part of the other effect. This was due to the fact that when a trend on the copy-numbers was simulated, individuals with 4 copies of the B allele were at higher risk than individuals with 3, 2, 1 or 0 copies, and inversely, when a trend on the number of B alleles was simulated, individuals with 4 copies were at higher risk than individuals with 3, 2, 1 or 0 copies. Thus the estimations provided by the *CN* and *Allele* (*multi*) strategies are biased (Text S1).

When a combined effect of the number of copies and of the number of B alleles was simulated (both RR_allele_>1 and RR_CN_>1), the most powerful strategy was always the *Joint* strategy testing for both effects, regardless of the strengths of the effects, the frequencies of both the B allele and the copy numbers, and the sample size (either 1,000 or 5,000 cases and controls). This pattern was observed when both deletions and duplication were simulated (Figure 2), as well as when only deletions (Figure 3) or duplications (Figure 4) were simulated (data not shown for 5,000 cases and 5,000 controls). Even in scenarios in which the power could approach 100%, we could observe that the *Joint* strategy yielded lower p-values for association (data not showed). Thus, we concluded that the best strategy showing the higher performances over all the investigated scenarios was the *Joint* strategy.

**Figure 5:**
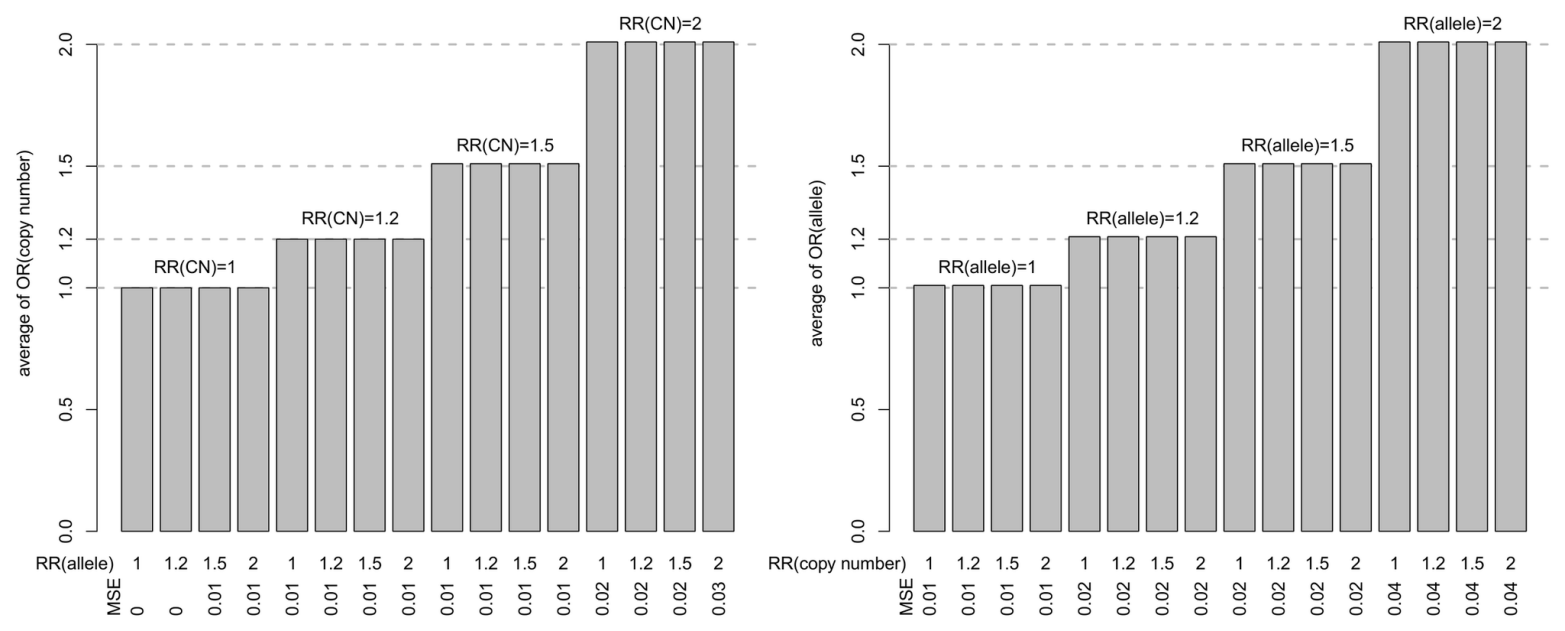
Estimations of the odds ratios using the *Joint* model. For each of the 16 scenarios of association strengths, this figure displays the average of the odds ratios for the effect of the copy number (left panel), and of the allele (right panel), obtained by transforming the *Joint* model coefficients. Averages were computed over 360,000 replicates (over all the 36 scenarios of frequencies and 10,000 replicates for each scenario) considering a sample of 1,000 cases and 1,000 controls.

In order to investigate whether the effects of the number of copies and the number of B alleles were well estimated, we then focused on the two coefficient terms in the model of the *Joint* strategy, the sum and the difference of the number of B and A alleles. We pointed out that the sum term provided an estimate of the copy number effect plus half of the B allele effect, whereas the difference term estimated half of the B allele effect (see Text S2). Thus, for each replicate of each scenario, we transformed both coefficient terms to get the estimation of the effects of both the B allele and the copy number. We then observed that, for each scenario, the medians of the estimated effects over the 10,000 replicates were exactly the simulated effects (Figure 5, Table S2). Thus, no bias was found in the estimation of both effects, regardless of the sample size (data not shown for 5,000 cases and 5,000 controls). However, as expected, the higher the sample-size, the lower the standard deviation of the estimated effects. It should be noted that the coefficients of the *CN* and the *Allele* (*multi*) strategies are estimates of the marginal risks of the number of copies and of the allele respectively and are thus biased estimates of the effect sizes (Text S1).

Next, we considered the situation where only the bi-allelic genotype information (AA, AB, BB or missing) was available as it is the case when investigators are interested in testing for association only with SNPs. To reconstruct the bi-allelic genotype information from the allele-specific copy-number states, we needed to evaluate the probabilities, given the allele-specific copy-number states, of the different bi-allelic genotypes. To estimate these probabilities, we used empirical data from the Spanish Bladder Cancer (SBC) / EPICURO study (see Material and Methods) showing that the SNP callings were almost never ambiguous when the number of copies was 1 or 2 and without almost missing data whereas the callings were really messy when the number of copies was 3, 4 or 0 with very high missing rates (Table S3). Interestingly, we observed that the probabilities of missing bi-allelic genotypes where not equal for the heterozygous allele-specific copy number states AAB and ABB in one hand, and AAAB and ABBB in the other hand (Table S3). This pattern could be due to a bias in the intensity measurements that tend to favor one of the two colors as was observed by Staaf et al. [18].

Because the bi-allelic dataset contained partial information on the allele and a high rate of missing genotypes, the association test of the effect of the B allele based on this information, evaluated through the *Allele* (*bi*) strategy, showed lower performances in comparison to the *Allele* (*multi*) strategy that tested for the same effect but using the allele-specific copy number states. This could be seen when both deletions and duplications were present (Figure 2), but also for deletions only (Figure 3), and duplications only (Figure 4 - data not shown for the dataset of 5,000 cases and 5,000 controls). Especially, when no effect of the B allele was simulated (RR_allele_=1 and RR_CN_>1), and regardless of the sample size, the power of the *Allele* (*bi*) strategy was null, meaning that the strategy could not detect the effect of the number of copies while the *Allele* (*multi*) strategy could (see the first columns of the Figures 2, 3 and 4). In this situation, the power of the *Allele* (*bi*) strategy had a tendency to decrease when the B allele frequency increased. This was due to the fact that a significant protective effect of the B allele was detected when the B allele frequency was low (data not showed). Regarding the effect estimation, we could observe that the effect of the B allele was under-estimated in most of the investigated scenarios, especially when deletions were simulated. Inversely, in the scenarios where duplications were simulated and the frequency of B was higher than 20%, the effect of the B allele tended to be overestimated (data not shown).

**Figure 6:**
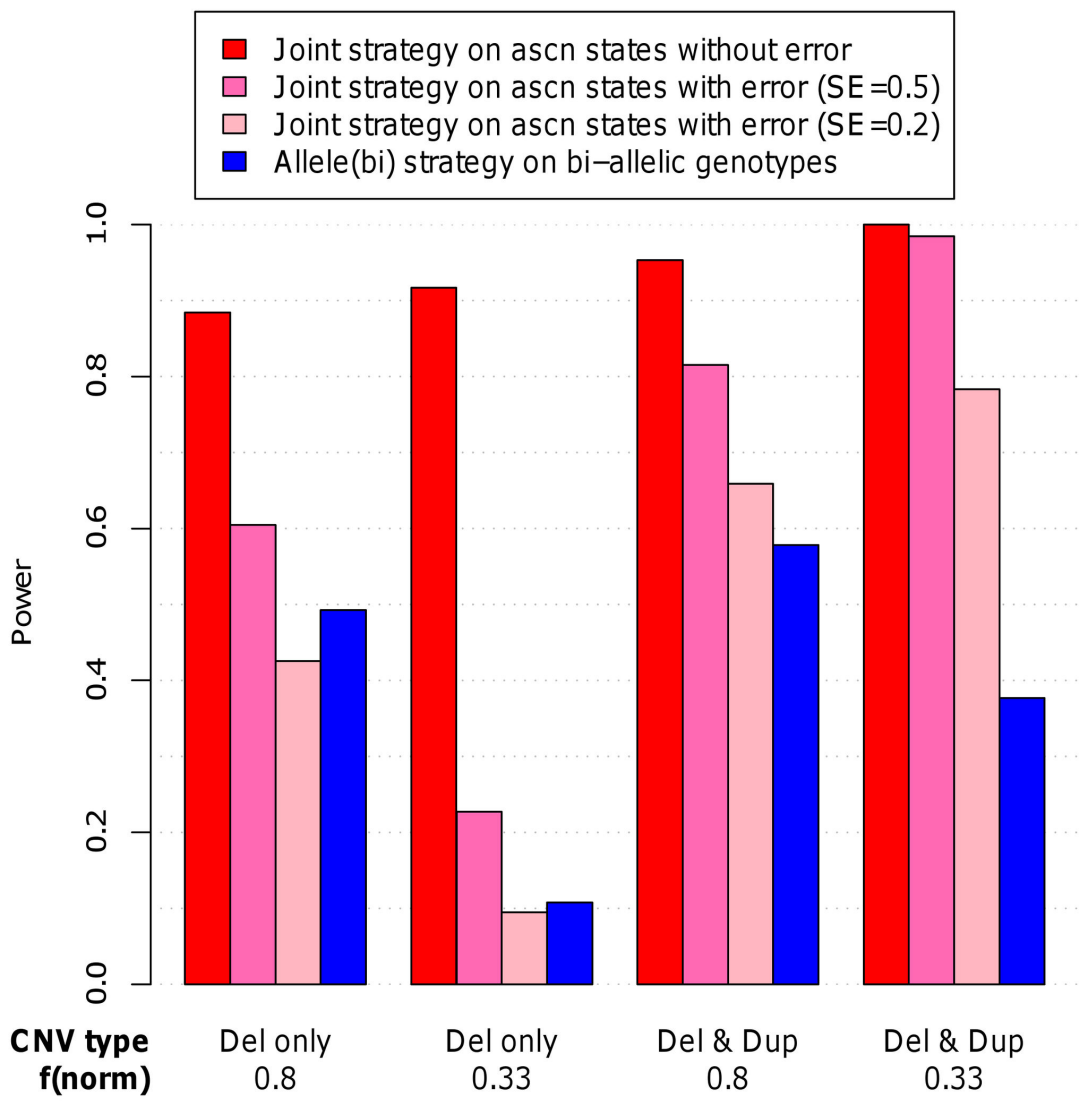
Power of the Joint strategy when errors are introduced in the allele-specific copy number states. This figure displays the power of the *Joint* strategy applied to allele-specific copy number states (ascn) without errors, of the *Joint* strategy applied to ascn states with errors due to a sensitivity (SE) of CNV detection reduced to 0.2 or 0.5 respectively and the power of the *Allele* (bi) strategy applied to the bi-allelic genotypes. The power was calculated using 10,000 replicates and considering 4 scenarios, all with a frequency of the B allele of 0.2, with relative risks of the number of copies and of the allele of 1.2 and a specificity of CNV detection of 0.99 for the ascn states with errors. Differences between the scenarios concern the type of CNV considered (deletions only (Del only) or both deletions and duplications (Del & Dup)) and the frequency of normal chromosomes carrying one copy of the CNV (f(norm) that was set to 0.33 or 0.80.

Next, and because the CNV calling can be unreliable as shown by the high discordance rates observed in duplicates [13], we introduced CNV calling errors (see the Material and Methods section for more details). As expected, the introduction of errors leads to a loss of power of the *Joint* strategy. This power loss was higher when the sensibility was lower and when the CNV types were deletions only. Interestingly, in the presence of both deletions and duplications, even with a sensitivity for CNV detection as low as 0.2, the power was still higher than the power of the *Allele* (*bi*) strategy using the bi-allelic genotypes in a SNP only analysis. When there were deletions only, depending on the sensitivity for CNV detection, the SNP only analysis using the *Allele* (*bi*) strategy could however perform better than the *Joint* strategy (Figure 6).

Finally, we applied the different methods on real CNV data from the HapMap project on chromosome 22 in 113 CEU individuals and 127 Yoruba (see Material and Methods). Based on the frequency of the allele and of the abnormal number of copies, we retained a total of 136 SNPs for the analysis (see Material and Methods). These SNPs appeared to be located in 3 copy-number regions in which the individual CNV breakpoints could be slightly different. Among these SNPs, 98, 90, 87 and 16 gave a significant result (after Bonferroni correction considering the total number of tests or the effective number of independent tests performed, see Material and Methods) with the *Joint*, the *Allele* (*multi*), the *Allele* (*bi*), and the *CN* strategies respectively (Figures S2 and S3, and Table S4). If we compare the different strategies, we found that there is good overlap in the results obtained using the *Joint*, the *Allele* (*multi*), and the *Allele* (*bi*) strategies except for 10 and 13 SNPs with a significant OR_allele_ in the *Joint* strategy that were not detected with the *Allele* (*multi*) and with the *Allele* (*bi*) strategies respectively. Both the *Allele* (*multi*) and the *Allele* (*bi*) strategies detected 2 SNPs that did not reach the Bonferroni-corrected significance level for the *Joint* strategy but were close to significance. The overlap in the results obtained using the *Joint* and the *CN* strategies was poor as shown in Figure S2, and the estimated OR_CN_ were very different. A striking example is SNP_A-8568797 with an estimated OR_CN_ of 0.42 with the *CN* strategy and 6.73 with the *Joint* strategy (Table S4). This important difference might be explained by the fact that, when looking at the overall population, the number of copies is indeed higher in controls than in cases (this explains the result obtained with the *CN* strategy). However, it is the opposite when looking within strata with a fixed number of A or B alleles and with allelic variation, i.e. within {A, AB, ABB}, {B, AB} and {BB, ABB} strata. In these strata, cases have more copies than controls (this explains the result obtained for the *Joint* strategy).

## Discussion

In this work, we investigated various strategies for association testing when a SNP is located within a CNV region. We simulated allele-specific copy number states for two different sample sizes of cases and controls (1,000 and 5,000), as well as the corresponding bi-allelic genotypes obtained in a classical SNP analysis in which all individuals are assumed to have two alleles. We considered a wide range of scenarios for both copy numbers and allele frequencies in relation to the tested strength of the association signal. We compared association testing strategies, either analyzing the effect of the allele, the effect of the number of copies, or both effects, either using the complete information of the alleles and the number of copies contained in the allele-specific copy number states or using the partial information on the allele only contained in the bi-allelic genotypes.

We found that, using the complete information of allele-specific copy number states, the model that performed best over all the considered scenarios was the one testing simultaneously for both the number of copies and the allele effects through two terms, the sum and the difference of the two allele counts, a model previously proposed by Korn et al., 2008 [11]. We showed that the statistical power of this model was better across a range of scenarios. We found that the coefficient terms of the model did not estimate the effects of the allele and the number of copies as suggested by Korn et al., 2008 [11], but that a transformation is needed to obtained unbiased estimations of these respective effects.

We also observed that testing the effect of the allele using partial information on the allele state contained in the bi-allelic genotypes led to a small loss of power in comparison to testing the effect of the allele using the complete information of allele and copy number contained in the allele-specific copy number states. This was true under most of the investigated scenarios. The B allele effect was not correctly estimated but was either under or over-estimated depending on the frequencies of deletions and duplications, respectively. Additionally, and on the basis of real data, we observed that, for a SNP located in a CNV region, the missing rate for bi-allelic genotypes was often quite high, and there were significant departures from Hardy-Weinberg equilibrium. Thus, and as previously mentioned [19], SNPs located in CNVs were likely to be excluded from classical SNP analysis and accordingly the test for the effect of the allele was not considered nor tested.

In this paper, we investigated various possibilities for common CNV frequency, from high (66%) to relatively low (20%), including various types of CNV, either deletions only, duplications only or both deletions and duplications. We also investigated a large range of possibilities for the SNP allele frequency, from rare (5%) to common (50%). Regarding the pattern of association, we were interested in both the effect of the copy number and the effect of the allele, thus, we simulated these two effects combined in an multiplicative manner, considering for each effect, the null effect of no association and various strengths of association, from small (relative risk of 1.2) to high (relative risk of 2). We believe these scenarios cover a wide range of possible situations. However there are also some limitations in the models we considered. In particular, we made the assumption that for a given allele-specific copy number state, for example BBB, the risk was the same for all possible combination of state on the two chromosomes (i.e., the risk was the same for an individual with two B alleles on one chromosome and one on the other than for an individual with three B alleles on one chromosome and zero on the other). Further work will be needed to investigate more complex scenarios and taking into account the repartition of the alleles across the two chromosomes, but this was beyond the scope of this paper.

By applying the studied strategies on HapMap chromosome 22 data, we were able to identify SNPs with numbers of copies and/or of the allele frequencies that varied between the CEU and Yoruba populations. Consistent with the simulations results, the *Joint* strategy was found to give more significant results than the *Allele* (*multi*) and *Allele* (*bi*) strategies, and few significant results were obtained with the *CN* strategy. It is true however that the *Joint strategy* can only be performed on a small proportion of the SNPs as it required that the studied SNPs fall within a common CNV. Of the 11,307 SNPs located in chromosome 22 in the HapMap data, only 136 were retained. It is thus difficult to compare the power of the classical test of association that will be performed on all the SNPs and the one of the Joint test that will only be performed on such a limited number of SNPs and will thus require a less stringent multiple testing correction. For these SNPs however that fall in common CNV and could be detected by using the *Joint* strategy, there is a risk that their effect on the disease will not be evidenced by the classical test and there is thus a gain of using the proposed strategy. Moreover, in the presence of both an effect of the number of copies and of the allele, effects will not be well estimated by the tests that only consider one of the effects. This was clear from the simulated and was also illustrated on the HapMap data where some SNPs showed very different OR_CN_ estimates with the *Joint* and the *CN* strategies, supporting the importance of considering jointly the effect of the allele and of the number of copies.

The calling of allele-specific copy number states needs a previous identification of the individual CNV regions to identify the total number of copies at each SNP. This CNV calling can be unreliable as shown by the high discordance rates observed in duplicates [13]. Nevertheless, we observed that, even with a low sensitivity of CNV detection, performing the *Joint* strategy using allele-specific copy number states is more powerful than performing a SNP-only analysis that does not take into account the total number of copies. With the availability of sequence data and the development of efficient algorithms to call allele and copy number with increasing accuracy, investigators are likely to become more and more interested in testing both effects and this work could then provide some insights into the statistical methodology to use.

## Materials and Methods

### Simulation design

We considered a SNP with two alleles A and B, B being the susceptibility allele, located within a CNV region that can be present in a variable number of copies, from 0 to 4. We simulated the allele-specific copy number states for this SNP for a set of 1,000 cases and 1,000 controls, as well as for a set of 5,000 cases and 5,000 controls, with various scenarios of genotype frequencies and inheritance models. Each scenario was replicated 10,000 times.

The frequency of each allele-specific copy number state in the control population was computed from the frequencies of each possible number of copies, from 0 to 4, and the frequencies of each possible allele, A and B (Table S5). On the basis that each individual had two chromosomes, we defined the frequencies of chromosomes carrying respectively 0 copy (deletion), 1 copy (normal) and 2 copies (duplication), through two simulation parameters: the frequency of chromosomes carrying one copy (f(norm) = {0.8, 0.5, 1/3}) and the type of CNV present in the population, either deletions only, duplications only or both. When a single type of CNV was present, the frequency of the deleted, respectively duplicated, chromosomes was f(del) = 1-f(norm) (respectively f(dup)=1-f(norm)). When both deletions and duplications were present, they were assumed to have equal probabilities to occur and the frequencies of both the deleted and duplicated chromosomes were f(del) = f(dup) = (1-f(norm))/2. Thus, the possible numbers of copies were from 0 to 4, with frequencies obtained by combining all possible pairs of chromosome leading to a particular number of copies (Table S5). The B allele frequency also varied from rare to common: f(B) = {0.05, 0.20, 0.35, 0.50}. In total, 36 different scenarios were investigated corresponding to different combinations of the parameters described above (Table S6).

The frequencies of the allele-specific copy number states in cases were calculated given the allele-specific copy number state frequencies in controls, and the relative risks (RRs) of the allele-specific copy number states versus the reference genotype (see Text S3). We chose the allele-specific copy number state AA as reference for the RRs. Because we were interested in a combined effect of the allele and the number of copies, we defined two RRs: RR_CN_ for the risk associated to an increase of 1 copy (reference is CN=2), and RR_allele_ for the risk associated to an increase of 1 B allele (reference is 0 B allele). Assuming that the allele and copy number effects were multiplicative, we derived the RR for each allele-specific copy number state as detailed in Table S5. RRs were varied in the range {1, 1.2, 1.5, 2}, which led to 16 scenarios of combined risks, including the null hypothesis scenario where both effects were set to 1.

In order to investigate the impact of performing a classical SNP analysis in which only information on bi-allelic genotypes or missing genotypes were available, we reconstructed from the simulated allele-specific copy number states, the bi-allelic genotypes. To estimate the probability of the different bi-allelic genotypes (AA, AB, BB and missing) given an allele-specific copy number state, we used real data from the Spanish Bladder Cancer (SBC) / EPICURO Study with more than 2000 individuals genotyped with the Illumina 1M array [20]. CNVs were called with PennCNV [12], and for each SNP located in a CNV region, the most probable allele-specific copy number state was determined. For instance, a SNP located in a 1-copy region can either be in the allele-specific copy number states A or B. If the BAF was lower than 0.5, the allele-specific copy number state was set to A and if the BAF was greater than 0.5, it was set to B. Since for these data we also had access to the bi-allelic genotype calls from Beadstudio, we could linked the allele-specific copy number state and the bi-allelic genotype call and determine for each allele-specific copy number state, the probabilities of being called AA, AB, BB or missing (Table S3).

Because the CNV detection sensitivity of SNP arrays is low, we also simulated allele-specific copy number states in which CNV calling errors were introduced. Based on previous works, we specified a sensitivity of 0.2 or 0.5 and a specificity of 0.99 for CNV detection [13,14]. For allele-specific copy number states with a total number of copies different from 2, we randomly decided with a probability of 0.2 or 0.5 whether the CNV was detected or not (sensitivity). In case the CNV was not detected, the allele-specific copy number state became a bi-allelic genotype. Namely, it became either AA or BB if the allele-specific copy number state was homozygous, or AB if heterozygous. And if the total number of copy was 0, it becames either AA, AB or BB with equal probabilities. For allele-specific copy number states with 2 copies, we randomly decided with a probability of 0.01 if a CNV was falsely detected (1-specificity). In case a CNV was falsely detected, we randomly decided of the number of copies, either 0, 1, 3 or 4, with equal probability. The allele-specific copy number state was then decided according to the original one (whether it was homozygous or not). Namely, if the allele-specific copy number state was AA, it either became A, AAA or AAAA for a wrong number of copies of 1, 3 or 4 respectively. If the allele-specific copy number state was BB, it either became B, BBB or BBBB for a wrong number of copies of 1, 3 or 4 respectively. If the allele-specific copy number state was AB, it became either A or B with equal probabilities for a wrong number of copies of 1, AAB or ABB for a wrong number of copies of 3, and AAAB, AABB or ABBB for a wrong number of copies of 4. In case the decision was a wrong zero copy detection, the allele-specific copy number state became the NULL state regardless of the initial state AA, AB or BB. On this more realistic dataset in which we introduced errors in the allele-specific copy number states, we performed the *Joint* strategy. In this analysis, we investigated 4 scenarios, the CNV type being either deletion only or both deletion and duplication, the frequency of normal chromosome carrying one copy of the CNV being either 0.33 or 0.8, the frequency of the B allele being 0.2 and the relative risks being 1.2.

### Association models

Allele and copy number effects were tested by different association strategies (Table 1). All these strategies used a likelihood-ratio test (LRT) comparing a logistic regression model to the null model. First we tested for the effect of the allele only assuming a trend on the number of B alleles. We used logistic regression models with the number of B as a continuous variable using either the allele-specific copy number states (*Allele* (*multi*) strategy) or the bi-allelic genotypes (*Allele* (*bi*) strategy). Second we tested for the effect of the number of copies only assuming a trend on the number of copies. We used a logistic regression model with the number of copies as a continuous variable (*CN* strategy). Finally, we used two logistic regression models that took into account the full information of allele and number of copies contained in the allele-specific copy number states. The first one was proposed by Korn et al. 2008 [11] and tested the association using a LRT with two degrees-of-freedom (*Joint* strategy). The two terms of the model are the sum and the difference of the number of copies of each allele. The second model is a general co-dominant model assessing the effect of each allele-specific copy number state (*Codominant* strategy), and testing for the association using a LRT whose the number of degrees-of-freedom depends on the number of observed allele-specific copy number states. In the *Joint* strategy, we then focused on both coefficients to detect an eventual bias in the estimations of the allele and copy number effects. For each scenario and each association strategy, the power was calculated as the percentage of replicates for which the p-value was lower than 0.05.

Simulations and statistical analysis were performed with R version 2.13.0 (http://www.r-project.org).

We provide some R functions to analyze case-control data with the *Joint* strategy and estimate odds ratios (Script S1). We also provide R functions to perform simulations and compare strategies (Script S2).

### HapMap data

We used publicly available genetic data from unrelated HapMap individuals from two populations, the CEU-CEPH population (CEU) and the Yoruba population (YOR). The genetic data were generated with the Affymetrix 6.0 SNP-array. We considered the individuals whose CEL files were available for download on the HapMap website (http://www.hapmap.org) and annotated as “included”, meaning the data were of good quality. We included 118 CEU individuals and 120 YOR individuals. The SNP calling was performed using Birdseed [11], and the CNV calling was performed using PennCNV and the PenCNV-affy module (http://www.openbioinformatics.org/penncnv/) [12]. We applied a CNV-specific quality control as recommended in the PennCNV documentation. Namely, we excluded from further analysis 5 CEU individuals and 3 YOR individuals with a standard deviation of the log R ratio greater than 0.32 or with a BAF drift greater than 0.0061. No individual were found to have a wave factor out of [-0.04; 0.04] and to have a BAF median out of [0.45; 0.55]. The analysis was restricted to the SNPs located on chromosome 22. Of the 11,307 SNPs on chromosome 22, we excluded the SNPs with a MAF < 0.05 (1,243 SNPs), the SNPs not located in CNV regions (10,773 SNPs) and the SNPs with a frequency of abnormal number of copies (different than 2) below 5% (380 SNPs). The allele-specific copy number states for the 136 remaining SNPs were computed using the R function provided in Script S1. The *Joint* strategy implemented in Script S1, as well as the *CN*, the *Allele* (*multi*), and the *Allele* (*bi*) strategies were run using the population as phenotype in the association analysis (CEU individuals as cases and YOR individuals as controls). The *Joint*, the *CN* and the *Allele* (*multi*) strategies were run on each of the 136 SNPs considered for this analysis, whereas the *Allele* (*bi*) strategy was run on a subset of 122 SNPs for which less than 5% of the genotypes were missing in the sample HapMap data. Bonferroni correction for multiple testing was applied for each strategy according to the respective number of tests. The *Joint* and the *Allele* (*multi*) strategies were corrected for 136 tests. The *Allele* (*bi*) strategy was corrected for 122 tests. Because the numbers of copies are highly correlated between SNPs, we applied a method similar to the method proposed by Li and Ji, 2005 [21] in order to calculate the effective number of tests to consider in the multiple testing correction for the *CN* strategy. The original method was based on the eigenvalues of the matrix of genotype correlation at the different markers. Here, we consider the matrix of correlation of the number of copies for the 136 SNPs instead. The effective number of independent test was estimated to be 16 and a Bonferroni correction for 16 tests was thus applied for the *CN* strategy.

## Supporting Information

Text S1 **Coefficients estimated in the *CN* and *Allele (multi)* strategies.**
(PDF)

Text S2 **Coefficient estimated in the *Joint* strategy.**
(PDF)

Text S3 **Calculation of the allele-specific copy number state frequencies in cases.**
(PDF)

Figure S1 **Type-1-errors.** Type-1-errors were estimated under the null hypothesis of no association, for the 36 investigated scenarios of frequencies, when simulating 1,000 cases and 1,000 controls (first row), and when simulating 5,000 cases and 5,000 controls (second row).
(PDF)

Figure S2 **Summary of the results of the association analysis on HapMap data.** This flowchart summarizes the results obtained by applying the *Joint*, the *CN*, the *Allele* (*multi*) and the *Allele* (*bi*) strategies to the chromosome 22 HapMap data.
(PDF)

Figure S3 **Manhattan and CNV plots of the association analysis on HapMap data.** The -log_10_ of the p-values of the *Joint* (panel **A**), the *CN* (panel **B**), the *Allele* (*multi*) (panel **C**) and the *Allele* (*bi*) (panel **D**) strategies are reported for the probes located in the 3 copy-number variant regions identified on chromosome 22 in the HapMap data. Panel **E** displays the detected CNVs in these 3 regions in the HapMap individuals used in the analysis - CEU as cases and YOR as controls - deletions are displayed in red and duplications in green.
(PDF)

Table S1 **Relative power of the *Joint* strategy.** For each of the 36 frequency scenarios investigated, this table displays the relative power of the *Joint* strategy, first relatively to the *CN* strategy when there is only and effect of the number of copies, and second relatively to the *Allele* (*multi*) strategy when there is only and effect of the allele.
(PDF)

Table S2 **Estimations of the coefficients and the odds ratios using the *Joint* model.** This table displays the means and the mean squared errors (MSE) of the *Joint* model coefficient estimates and of the odds ratios for the allele and copy number effect after transforming the *Joint* model coefficients, computed for each of the 16 scenarios of association strengths, over 360,000 replicates (the 10,000 replicates of each scenario and all the 36 scenarios of frequencies) considering a sample of 1,000 cases and 1,000 controls.
(PDF)

Table S3 **Bi-allelic genotype probabilities.**
(PDF)

Table S4 **Results of the association analysis on HapMap data.** This table displays the results of the *Joint*, the *CN*, the *Allele* (*multi*) and the *Allele* (*bi*) strategies applied to HapMap data, restricting the analysis to the chromosome 22. The SNPs excluded from the *Allele* (*bi*) strategies because of a missing rate greater than 5% have no results for this strategy (displayed with “-“).
(XLSX)

Table S5 **Details of the computation of the allele-specific copy number states frequencies and relative risks.** The notations f(del), f(norm) and f(dup) refer to the frequencies of chromosomes carrying respectively 0, 1 and 2 copies of the CNV, f(B) is the frequency of the allele B. RR_allele|CN_ and RR_CN|allele_ are the relative risks associated respectively to an increase of one allele B, and an increase of one copy.
(PDF)

Table S6 **Expected allele-specific copy number states frequencies.** The frequencies are displayed for each of the 36 frequency scenarios investigated.
(PDF)

Script S1 **R scripts implementing the computation of the allele-specific copy number states and the association analysis using the *Joint* strategy.** This compressed file contains 1- an R script (AssociationSNPinCNV.R), 2- a manual describing the arguments and values of the R functions implemented in AssociationSNPinCNV.R (Manual AssociationSNPinCNV.txt), 3- a tutorial (Tutorial AssociationSNPinCNV.txt) and input files (DataProbes.txt, penncnv.rawcnv, BAFtable.txt, DataIndividuals.txt) as an example to run the functions implemented in the R script AssociationSNPinCNV.R.
(ZIP)

Script S2 **R scripts to simulate and analyze allele-specific copy number states and bi-allelic genotypes for datasets of cases and controls.** This compressed file contains 1- an R script (ASCNsimulationsAndAssociationStrategies.R), 2- a manual describing the arguments and values of the R functions implemented in ASCNsimulationsAndAssociationStrategies.R (Manual ASCNsimulationsAndAssociationStrategies.txt), 3- a tutorial as an example to run the functions implemented in the R script ASCNsimulationsAndAssociationStrategies.R (Tutorial ASCNsimulationsAndAssociationStrategies.txt).
(ZIP)
