# Supplementary material for: Advantage of Using Allele-Specific Copy Numbers When Testing for Association in Regions with Common Copy Number Variants
Source: PLoS One. 2013 Sep 10;8(9):e75350. doi: 10.1371/journal.pone.0075350 (PMC3769257; doi:10.1371/journal.pone.0075350)
Supplement: Text S1 — Coefficients estimated in the CN and Allele (multi) strategies. (PDF) [file pone.0075350.s001.pdf]

Text S1. Coefficients estimated in the *CN* and *Allele (multi)* strategies.

Let CN being the number of copies, b being the number of allele B, A^iB^j being the allele-specific copy number state with i alleles A and j alleles B, $f(B)$ being the frequency of the allele B, $f(CN=i)$ being the frequency of i number of copies.

RR_{CN} is the effect of the number of copies estimated by the *CN* strategy that does not take into account the effect of the allele. For n varying between 0 and 3:

$$RR_{CN} = RR_{CN=n+1}/_{CN=n} = \frac{P(D^+|CN = n+1)}{P(D^+|CN = n)}$$

$$RR_{CN} = \frac{P\left(D^+\middle|\{A^{n+1-k}B^k\,;\ k = 0,..,n+1\}\right)}{P(D^+|\{A^{n-k}B^k\,;\ k = 0,..,n\})} * \frac{P(D^+|AA)}{P(D^+|AA)}$$

$$RR_{CN} = \frac{\sum_{k=0}^{k=n+1} C_{n+1}^k f(B)^k (1-f(B))^{n+1-k} RR_{allele|CN}^k RR_{CN|allele}^{n+1-2}}{\sum_{k=0}^{k=n} C_n^k f(B)^k (1-f(B))^{n-k} RR_{allele|CN}^k RR_{CN|allele}^{n-2}}$$

$$RR_{CN} = \frac{RR_{CN|allele}^{n+1-2}}{RR_{CN|allele}^{n-2}} * \frac{\left(1-f(B)+f(B)RR_{allele|CN}\right)^{n+1}}{\left(1-f(B)+f(B)RR_{allele|CN}\right)^{n}}$$

$$RR_{CN} = RR_{CN|allele} * \left(1-f(B)+f(B)RR_{allele|CN}\right)$$

Thus, for no effect of the allele, the relative risk of the number of copies estimated by the *CN* strategy is unbiased:

$$RR_{allele|CN} = 1 \Rightarrow RR_{CN} = RR_{CN|allele}$$

RR_{allele} is the effect of the allele estimated by the *Allele (multi)* strategy that does not take into account the effect of the number of copies.

$$RR_{allele} = RR_{b=n+1}/_{b=n} = \frac{P(D^+|b = n+1)}{P(D^+|b = n)}$$

$$RR_{allele} = \frac{P\left(D^+\middle|\{A^kB^{n+1}\,;\ k = 0,..,4-n-1\}\right)}{P(D^+|\{A^kB^n\,;\ k = 0,..,4-n\})} * \frac{P(D^+|AA)}{P(D^+|AA)}$$

$$RR_{allele} = \frac{\sum_{k=0}^{k=4-n-1} \lambda_{A^kB^{n+1}} RR_{allele|CN}^{n+1} RR_{CN|allele}^{k+n+1-2}}{\sum_{k=0}^{k=4-n} \lambda_{A^kB^n} RR_{allele|CN}^{n} RR_{CN|allele}^{k+n-2}}$$

$$RR_{allele} = RR_{allele|CN} * \frac{\sum_{k=0}^{k=4-n-1} \lambda_{A^kB^{n+1}} RR_{CN|allele}^{k+n+1-2}}{\sum_{k=0}^{k=4-n} \lambda_{A^kB^n} RR_{CN|allele}^{k+n-2}}$$

Where $\lambda_{A^kB^n}$ is the frequency of the allele-specific copy number state A^kB^n within the subset of the allele-specific copy number states carrying n alleles B $\{A^kB^n\,;\ k = 0,..,4-n\}$.

$\lambda_{A^k B^n}$ depends on the frequency of B and the frequency of the number of copies. The frequency of the number of copies is not a parameter of the simulations, rather it depends on the frequency of normal chromosome carrying one copy of the CNV (parameter f(norm) in the simulations) and on the CNV type.

$$\lambda_{A^k B^n} = \frac{C_{n+k}^{k} f(CN = n + k) f(B)^n (1 - f(B))^k}{\sum_{k=0}^{k=4-n} C_{n+k}^{k} f(CN = n + k) f(B)^n (1 - f(B))^k}$$

$$\sum_{k=0}^{k=4-n} \lambda_{A^k B^n} = 1$$

Thus, for no effect of the number of copies, the relative risk of the allele estimated by the *Allele (multi)* strategy is unbiased:

$$RR_{CN|allele} = 1 \Rightarrow RR_{allele} = RR_{allele|CN}$$
