# Supplementary material for: Advantage of Using Allele-Specific Copy Numbers When Testing for Association in Regions with Common Copy Number Variants
Source: PLoS One. 2013 Sep 10;8(9):e75350. doi: 10.1371/journal.pone.0075350 (PMC3769257; doi:10.1371/journal.pone.0075350)
Supplement: Text S2 — Coefficient estimated in the Joint strategy. (PDF) [file pone.0075350.s002.pdf]

Text S2. Coefficient estimated in the *Joint* strategy.

The joint model is logit(p) ~ $\alpha + \beta_1(B+A) + \beta_2(B-A)$, in which A and B are respectively the number of A alleles and the number of B alleles.

Each of the two coefficients is adjusted on the other coefficient, thus the odds ratios (OR) are adjusted or conditioned on the other coefficient that is fixed.

$$OR_{B+A|B-A} = e^{\beta_1}$$

$$OR_{B-A|B+A} = e^{\beta_2}$$

The odds ratio associated to the difference in allelic counts in the allelic-specific copy-number states (B-A) is written as followed:

$$OR_{B-A|B+A} = \frac{P(D^+|B+A=s, B-A=d+1)}{P(D^+|B+A=s, B-A=d)} \frac{P(D^-|B+A=s, B-A=d)}{P(D^-|B+A=s, B-A=d+1)}$$

We can transformed the conditional part as followed

$$P(X|B+A=s, B-A=d+1) = P\left(X\middle|B+A=s, B=b+\tfrac{1}{2}\right)$$

$$P(X|B+A=s, B-A=d) = P(X|B+A=s, B=b)$$

$$b = \tfrac{1}{2}(d+s)$$

Thus, we can write

$$OR_{B-A|B+A} = \frac{P\left(D^+\middle|B+A=s, B=b+\frac{1}{2}\right)}{P(D^+|B+A=s, B=b)} \frac{P(D^-|B+A=s, B=b)}{P\left(D^-\middle|B+A=s, B=b+\frac{1}{2}\right)}$$

$$\boxed{OR_{B-A|B+A} = \left(OR_{allele|CN}\right)^{1/2}}$$

In which $OR_{allele|CN}$ corresponds to the odds ratio of an extra copy of the B allele, for a fixed number of copies. This odds ratio is a trend on the continuous variable that is the number of B alleles, thus, by definition, an increase of half of this value leads to a risk equivalent to the square of the trend odds ratio (Breslow and Day, 1980).

The odds ratio associated to the total copy-number (B+A) is written as followed:

$$OR_{B+A|B-A} = \frac{P(D^+|B+A=s+1, B-A=d)}{P(D^+|B+A=s, B-A=d)} \frac{P(D^-|B+A=s, B-A=d)}{P(D^-|B+A=s+1, B-A=d)}$$

The conditional part can be transformed as followed

$$P(X|B+A=s+1, B-A=d) = P\left(X\middle|B+A=s+1, B=b+\tfrac{1}{2}\right)$$

$$P(X|B+A=s, B-A=d) = P(X|B+A=s, B=b)$$

$$b = \tfrac{1}{2}(d+s)$$

And thus,

$$OR_{B+A|B-A} = \frac{P\left(D^+\middle|B+A=s+1, B=b+\frac{1}{2}\right)}{P(D^+|B+A=s, B=b)} \frac{P(D^-|B+A=s, B=b)}{P\left(D^-\middle|B+A=s+1, B=b+\frac{1}{2}\right)}$$

$$OR_{B+A|B-A} = \frac{P\left(D^+\middle|B+A=s+1, B=b+\frac{1}{2}\right)}{P(D^+|B+A=s+1, B=b)} \times \frac{P(D^+|B+A=s+1, B=b)}{P(D^+|B+A=s, B=b)} \times \frac{P(D^-|B+A=s, B=b)}{P(D^-|B+A=s+1, B=b)} \times \frac{P(D^-|B+A=s+1, B=b)}{P\left(D^-\middle|B+A=s+1, B=b+\frac{1}{2}\right)}$$

$$OR_{B+A|B-A} = \frac{P\left(D^+\middle|B+A=s+1, B=b+\frac{1}{2}\right)}{P(D^+|B+A=s+1, B=b)} \times \frac{P(D^-|B+A=s+1, B=b)}{P\left(D^-\middle|B+A=s+1, B=b+\frac{1}{2}\right)} \times \frac{P(D^+|B+A=s+1, B=b)}{P(D^+|B+A=s, B=b)} \times \frac{P(D^-|B+A=s, B=b)}{P(D^-|B+A=s+1, B=b)}$$

$$\boxed{OR_{B+A|B-A} = \left(OR_{allele|CN}\right)^{1/2} \times OR_{CN|allele}}$$

In which $OR_{allele|CN}$ corresponds to the odds ratio of an extra copy of the B allele, for a fixed number of copies; and $OR_{CN|allele}$ corresponds to the odds ratio of an extra copy of the CNV, for a fixed number of B allele.

Breslow, N. E. and N. E. Day (1980). "Statistical methods in cancer research. Volume I - The analysis of case-control studies." IARC Sci Publ(32): 5-338.
