# Supplementary material for: Advantage of Using Allele-Specific Copy Numbers When Testing for Association in Regions with Common Copy Number Variants
Source: PLoS One. 2013 Sep 10;8(9):e75350. doi: 10.1371/journal.pone.0075350 (PMC3769257; doi:10.1371/journal.pone.0075350)
Supplement: Text S3 — Calculation of the allele-specific copy number state frequencies in cases. (PDF) [file pone.0075350.s003.pdf]

Text S3. Calculation of the allele-specific copy number state frequencies in cases.

The expected frequency of the allele-specific copy number state g in cases $P(g|D^{+})$ depends on the expected frequencies of allele-specific copy number state g in controls and the relative risk of g (RR_g).

$$P(g|D^{+}) = \frac{P(D^{+}|g)\times P(g)}{P(D^{+})} = \frac{P(D^{+}|g)\times P(g)}{\sum_i P(D^{+}|g_i)\times P(g_i)} = \frac{RR_g\times P(g)}{\sum_i RR_{g_i}\times P(g_i)}$$
