# Supplementary material for: Advantage of Using Allele-Specific Copy Numbers When Testing for Association in Regions with Common Copy Number Variants
Source: PLoS One. 2013 Sep 10;8(9):e75350. doi: 10.1371/journal.pone.0075350 (PMC3769257; doi:10.1371/journal.pone.0075350)

Figure S1. **Type-1-errors.** Type-1-errors were estimated under the null hypothesis of no association, for the 36 investigated scenarios of frequencies, when simulating 1,000 cases and 1,000 controls (first row), and when simulating 5,000 cases and 5,000 controls (second row).

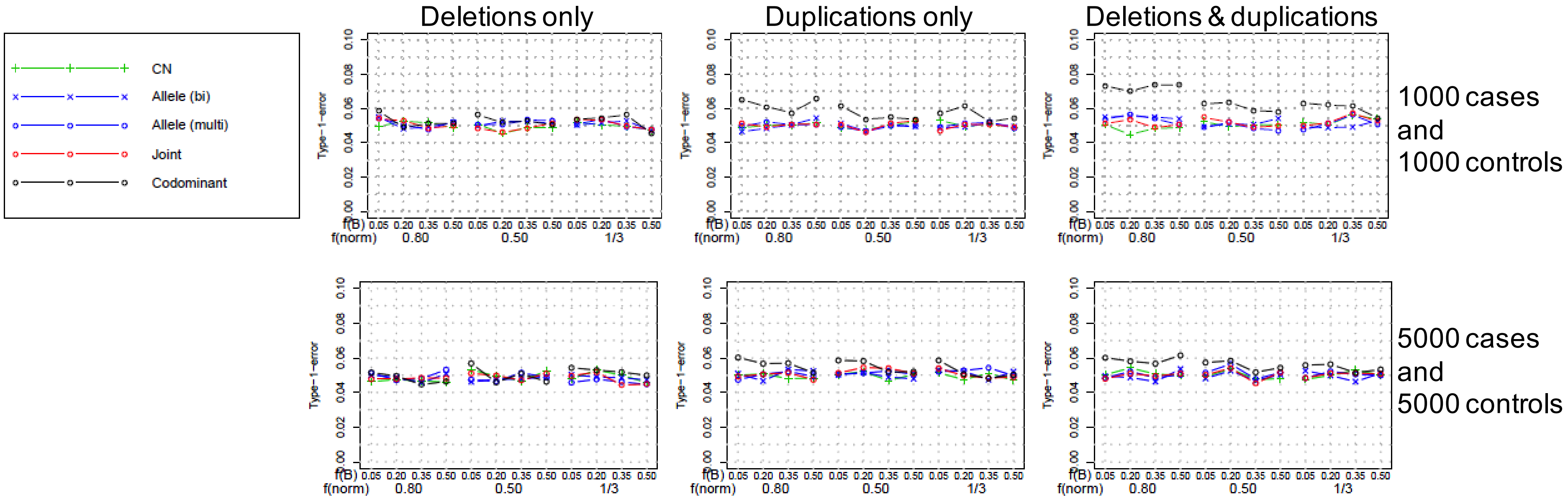

Supplement: Figure S1 — Type-1-errors. Type-1-errors were estimated under the null hypothesis of no association, for the 36 investigated scenarios of frequencies, when simulating 1,000 cases and 1,000 controls (first row), and when simulating 5,000 cases and 5,000 controls (second row). (PDF) [file pone.0075350.s004.pdf]
