# Supplementary material for: Advantage of Using Allele-Specific Copy Numbers When Testing for Association in Regions with Common Copy Number Variants
Source: PLoS One. 2013 Sep 10;8(9):e75350. doi: 10.1371/journal.pone.0075350 (PMC3769257; doi:10.1371/journal.pone.0075350)
Supplement: Figure S2 — Summary of the results of the association analysis on HapMap data. This flowchart summarizes the results obtained by applying the Joint, the CN, the Allele (multi) and the Allele (bi) strategies to the chromosome 22 HapMap data. (PDF) [file pone.0075350.s005.pdf]

Figure S2. **Summary of the results of the association analysis on HapMap data.** This flowchart summarizes the results obtained by applying the *Joint*, the *CN*, the *Allele (multi)* and the *Allele (bi)* strategies to the chromosome 22 HapMap data.

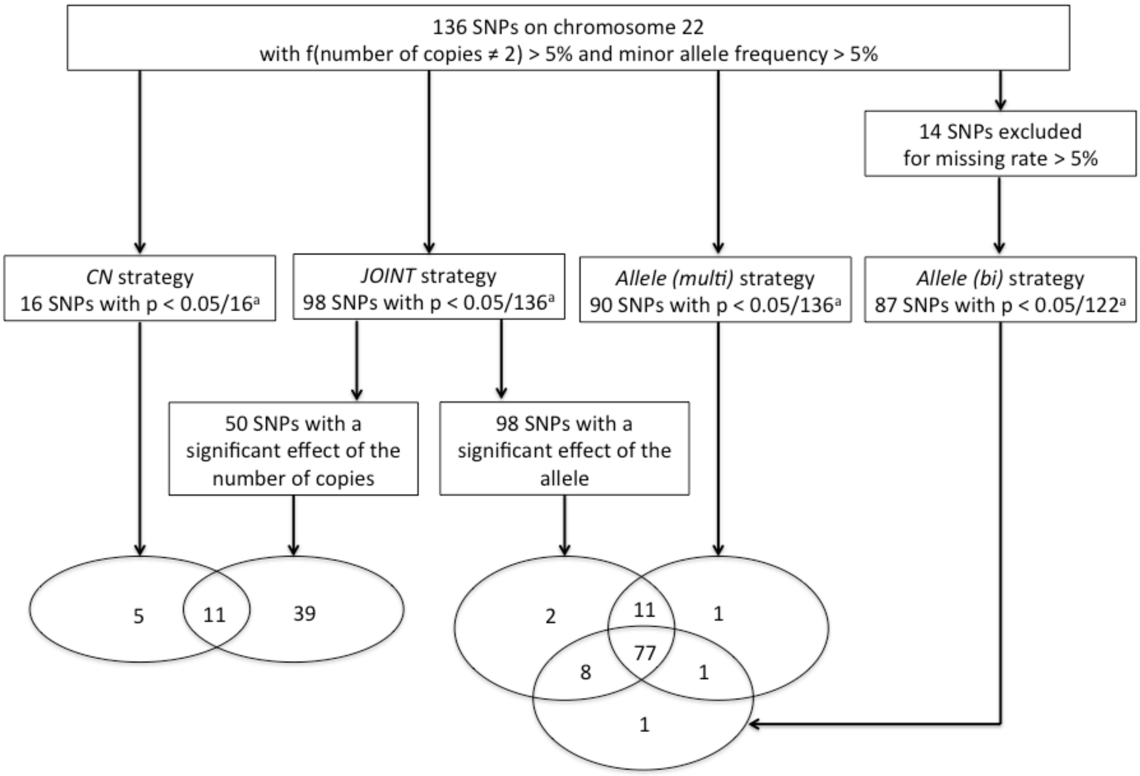

[a] p refers to the p-value of the likelihood ratio test. Bonferroni correction for multiple testing was applied for each strategy according to the number of tests (or to the effective number of tests for the CN strategy - see Material and Methods).
