# Supplementary material for: Advantage of Using Allele-Specific Copy Numbers When Testing for Association in Regions with Common Copy Number Variants
Source: PLoS One. 2013 Sep 10;8(9):e75350. doi: 10.1371/journal.pone.0075350 (PMC3769257; doi:10.1371/journal.pone.0075350)

Figure S3. Manhattan and CNV plots of the association analysis on HapMap data. The –log10 of the p-values of the *Joint* (panel **A**), the *CN* (panel **B**), the *Allele (multi)* (panel **C**) and the *Allele (bi)* (panel **D**) strategies are reported for the probes located in the 3 copy-number variant regions identified on chromosome 22 in the HapMap data. Panel **E** displays the detected CNVs in these 3 regions in the HapMap individuals used in the analysis - CEU as cases and YOR as controls - deletions are displayed in red and duplications in green.

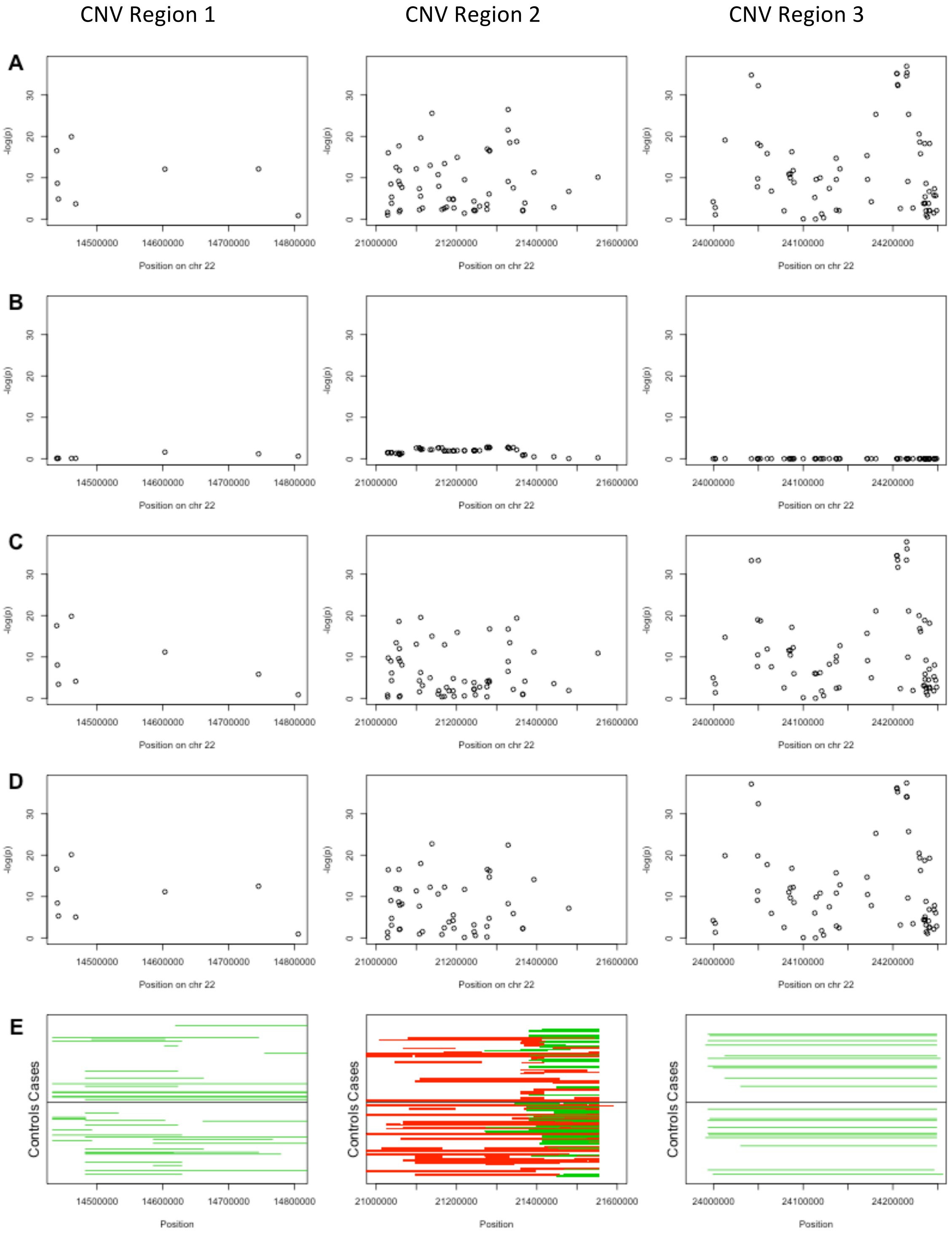

Supplement: Figure S3 — Manhattan and CNV plots of the association analysis on HapMap data. The -log10 of the p-values of the Joint (panel A), the CN (panel B), the Allele (multi) (panel C) and the Allele (bi) (panel D) strategies are reported for the probes located in the 3 copy-number variant regions identified on chromosome 22 in the HapMap data. Panel E displays the detected CNVs in these 3 regions in the HapMap individuals used in the analysis - CEU as cases and YOR as controls - deletions are displayed in red and duplications in green. (PDF) [file pone.0075350.s006.pdf]
