# Supplementary material for: Advantage of Using Allele-Specific Copy Numbers When Testing for Association in Regions with Common Copy Number Variants
Source: PLoS One. 2013 Sep 10;8(9):e75350. doi: 10.1371/journal.pone.0075350 (PMC3769257; doi:10.1371/journal.pone.0075350)
Supplement: Table S1 — Relative power of the Joint strategy. For each of the 36 frequency scenarios investigated, this table displays the relative power of the Joint strategy, first relatively to the CN strategy when there is only and effect of the number of copies, and second relatively to the Allele (multi) strategy when there is only and effect of the allele. (PDF) [file pone.0075350.s007.pdf]

Table S1. **Relative power of the *Joint* strategy.** For each of the 36 frequency scenarios investigated, this table displays the relative power of the *Joint* strategy, first relatively to the *CN* strategy when there is only and effect of the number of copies, and second relatively to the *Allele (multi)* strategy when there is only and effect of the allele.

			Relative power to the *CN* strategy when effect of the number of copies only (RR_{allele}=1)			Relative power to the A*llele(multi)* strategy when effect of the allele only (RR_{CN}=1)		
f(B)	CNVtype	f(norm)	RR_{CN}=1.2	RR_{CN}=1.5	RR_{CN}=2	RR_{allele}=1.2	RR_{allele}=1.5	RR_{allele}=2
**0.05**	**Deletions**	**0.8**	0.83	1.00	1.00	0.76	0.88	1.00
		**0.5**	0.90	1.00	1.00	0.76	0.82	0.98
		**1/3**	0.88	1.00	1.00	0.83	0.79	0.94
	**Del & Dup**	**0.8**	0.86	1.00	1.00	0.76	0.92	1.00
		**0.5**	0.98	1.00	1.00	0.79	0.92	1.00
		**1/3**	0.99	1.00	1.00	0.78	0.92	1.00
	**Duplications**	**0.8**	0.84	1.00	1.00	0.77	0.94	1.00
		**0.5**	0.90	1.00	1.00	0.78	0.97	1.00
		**1/3**	0.88	1.00	1.00	0.78	0.97	1.00
**0.20**	**Deletions**	**0.8**	0.83	1.00	1.00	0.83	1.00	1.00
		**0.5**	0.89	1.00	1.00	0.78	0.98	1.00
		**1/3**	0.88	1.00	1.00	0.77	0.94	1.00
	**Del & Dup**	**0.8**	0.86	1.00	1.00	0.84	1.00	1.00
		**0.5**	0.98	1.00	1.00	0.85	1.00	1.00
		**1/3**	0.99	1.00	1.00	0.87	1.00	1.00
	**Duplications**	**0.8**	0.84	1.00	1.00	0.87	1.00	1.00
		**0.5**	0.90	1.00	1.00	0.90	1.00	1.00
		**1/3**	0.87	1.00	1.00	0.92	1.00	1.00
**0.35**	**Deletions**	**0.8**	0.83	1.00	1.00	0.87	1.00	1.00
		**0.5**	0.90	1.00	1.00	0.83	1.00	1.00
		**1/3**	0.88	1.00	1.00	0.80	0.99	1.00
	**Del & Dup**	**0.8**	0.86	1.00	1.00	0.90	1.00	1.00
		**0.5**	0.98	1.00	1.00	0.92	1.00	1.00
		**1/3**	1.00	1.00	1.00	0.93	1.00	1.00
	**Duplications**	**0.8**	0.83	1.00	1.00	0.92	1.00	1.00
		**0.5**	0.89	1.00	1.00	0.96	1.00	1.00
		**1/3**	0.88	1.00	1.00	0.96	1.00	1.00
**0.50**	**Deletions**	**0.8**	0.82	1.00	1.00	0.89	1.00	1.00
		**0.5**	0.89	1.00	1.00	0.86	1.00	1.00
		**1/3**	0.88	1.00	1.00	0.83	1.00	1.00
	**Del & Dup**	**0.8**	0.86	1.00	1.00	0.92	1.00	1.00
		**0.5**	0.98	1.00	1.00	0.96	1.00	1.00
		**1/3**	0.99	1.00	1.00	0.97	1.00	1.00
	**Duplications**	**0.8**	0.83	1.00	1.00	0.94	1.00	1.00
		**0.5**	0.89	1.00	1.00	0.97	1.00	1.00
		**1/3**	0.87	1.00	1.00	0.98	1.00	1.00
