# Supplementary material for: Advantage of Using Allele-Specific Copy Numbers When Testing for Association in Regions with Common Copy Number Variants
Source: PLoS One. 2013 Sep 10;8(9):e75350. doi: 10.1371/journal.pone.0075350 (PMC3769257; doi:10.1371/journal.pone.0075350)
Supplement: Table S2 — Estimations of the coefficients and the odds ratios using the Joint model. This table displays the means and the mean squared errors (MSE) of the Joint model coefficient estimates and of the odds ratios for the allele and copy number effect after transforming the Joint model coefficients, computed for each of the 16 scenarios of association strengths, over 360,000 replicates (the 10,000 replicates of each scenario and all the 36 scenarios of frequencies) considering a sample of 1,000 cases and 1,000 controls. (PDF) [file pone.0075350.s008.pdf]

**Table S2**. **Estimations of the coefficients and the odds ratios using the *Joint* model.** This table displays the means and the mean squared errors (MSE) of the *Joint* model coefficient estimates and of the odds ratios for the allele and copy number effect after transforming the *Joint* model coefficients, computed for each of the 16 scenarios of association strengths, over 360,000 replicates (the 10,000 replicates of each scenario and all the 36 scenarios of frequencies) considering a sample of 1,000 cases and 1,000 controls.

| Simulated relative risks | | Coefficients in the model | | | | Estimated odds ratios after transformation | | | |
|---|---|---|---|---|---|---|---|---|---|
| | | difference term | | sum term | | copy number | | allele | |
| $RR_{CN}$ | $RR_{allele}$ | mean | MSE | mean | MSE | mean | MSE | mean | MSE |
| 1 | 1 | 0.00 | 0.00 | 0.00 | 0.01 | 1.00 | 0.00 | 1.01 | 0.01 |
| 1 | 1.2 | 0.09 | 0.00 | 0.09 | 0.01 | 1.00 | 0.00 | 1.21 | 0.02 |
| 1 | 1.5 | 0.20 | 0.00 | 0.20 | 0.01 | 1.00 | 0.01 | 1.51 | 0.02 |
| 1 | 2 | 0.35 | 0.00 | 0.35 | 0.01 | 1.00 | 0.01 | 2.01 | 0.04 |
| 1.2 | 1 | 0.00 | 0.00 | 0.18 | 0.01 | 1.20 | 0.01 | 1.01 | 0.01 |
| 1.2 | 1.2 | 0.09 | 0.00 | 0.27 | 0.01 | 1.20 | 0.01 | 1.21 | 0.02 |
| 1.2 | 1.5 | 0.20 | 0.00 | 0.39 | 0.01 | 1.20 | 0.01 | 1.51 | 0.02 |
| 1.2 | 2 | 0.35 | 0.00 | 0.53 | 0.01 | 1.20 | 0.01 | 2.01 | 0.04 |
| 1.5 | 1 | 0.00 | 0.00 | 0.41 | 0.01 | 1.51 | 0.01 | 1.01 | 0.01 |
| 1.5 | 1.2 | 0.09 | 0.00 | 0.50 | 0.01 | 1.51 | 0.01 | 1.21 | 0.02 |
| 1.5 | 1.5 | 0.20 | 0.00 | 0.61 | 0.01 | 1.51 | 0.01 | 1.51 | 0.02 |
| 1.5 | 2 | 0.35 | 0.00 | 0.75 | 0.01 | 1.51 | 0.01 | 2.01 | 0.04 |
| 2 | 1 | 0.00 | 0.00 | 0.69 | 0.01 | 2.01 | 0.02 | 1.01 | 0.01 |
| 2 | 1.2 | 0.09 | 0.00 | 0.79 | 0.01 | 2.01 | 0.02 | 1.21 | 0.02 |
| 2 | 1.5 | 0.20 | 0.00 | 0.90 | 0.01 | 2.01 | 0.02 | 1.51 | 0.02 |
| 2 | 2 | 0.35 | 0.00 | 1.04 | 0.01 | 2.01 | 0.03 | 2.01 | 0.04 |
