# Supplementary material for: Advantage of Using Allele-Specific Copy Numbers When Testing for Association in Regions with Common Copy Number Variants
Source: PLoS One. 2013 Sep 10;8(9):e75350. doi: 10.1371/journal.pone.0075350 (PMC3769257; doi:10.1371/journal.pone.0075350)
Supplement: Table S3 — Bi-allelic genotype probabilities. (PDF) [file pone.0075350.s009.pdf]

Table S3. Bi-allelic genotype probabilities.

allele-specific copy number states		Probabilities of bi-allelic genotypes			
		AA	AB	BB	Missing
**CN=0**	**NULL**	5%	20%	5%	70%
**CN=1**	**A**	99%	-	-	1%
	**B**	-	-	99%	1%
**CN=2**	**AA**	100%	-	-	-
	**AB**	-	100%	-	
	**BB**	-	-	100%	-
**CN=3**	**AAA**	100%	-	-	-
	**AAB**	-	55%	-	45%
	**ABB**	-	90%	-	10%
	**BBB**	-	-	100%	-
**CN=4**	**AAAA**	100.00%	-	-	-
	**AAAB**	-	8.00%	-	92.00%
	**AABB**	-	100.00%	-	-
	**ABBB**	-	35.00%	-	65.00%
	**BBBB**	-	-	100.00%	-
