# Supplementary material for: Advantage of Using Allele-Specific Copy Numbers When Testing for Association in Regions with Common Copy Number Variants
Source: PLoS One. 2013 Sep 10;8(9):e75350. doi: 10.1371/journal.pone.0075350 (PMC3769257; doi:10.1371/journal.pone.0075350)
Supplement: Table S5 — Details of the computation of the allele-specific copy number states frequencies and relative risks. The notations f(del), f(norm) and f(dup) refer to the frequencies of chromosomes carrying respectively 0, 1 and 2 copies of the CNV, f(B) is the frequency of the allele B. RRallele|CN and RRCN|allele are the relative risks associated respectively to an increase of one allele B, and an increase of one copy. (PDF) [file pone.0075350.s011.pdf]

Table S5. Details of the computation of the allele-specific copy number states frequencies and relative risks. The notations f(del), f(norm) and f(dup) refer to the frequencies of chromosomes carrying respectively 0, 1 and 2 copies of the CNV, f(B) is the frequency of the allele B. $RR_{allele|CN}$ and $RR_{CN|allele}$ are the relative risks associated respectively to an increase of one allele B, and an increase of one copy.

Calculation of frequencies and relative risks				
**Numbers of copies**		**allele-specific copy number states**		
	**frequencies**		**frequencies**	**RRs (versus AA)**
**CN=0**	f(0)=f(del)*f(del)	**NULL**	f(0)	$1/(RR_{CN\|allele})^2$
**CN=1**	f(1)=2*f(del)*f(norm)	**A**	f(1)*(1-f(B))	$1/RR_{CN\|allele}$
		**B**	f(1)*f(B)	$RR_{allele\|CN}/RR_{CN\|allele}$
**CN=2**	f(2)=2*f(del)*f(dup) + f(norm)^2	**AA**	f(2)*(1-f(B))^2	**1 (reference)**
		**AB**	f(2)*2*f(B)*(1-f(B))	$RR_{allele\|CN}$
		**BB**	f(2)*f(B)^2	$(RR_{allele\|CN})^2$
**CN=3**	f(3)=2*f(dup)*f(norm)	**AAA**	f(3)*(1-f(B))^3	$RR_{CN\|allele}$
		**AAB**	f(3)*3*f(B)*(1-f(B))^2	$RR_{allele\|CN}*RR_{CN\|allele}$
		**ABB**	f(3)*3*(1-f(B))*f(B)^2	$(RR_{allele\|CN})^2*RR_{CN\|allele}$
		**BBB**	f(3)*f(B)^3	$(RR_{allele\|CN})^3*RR_{CN\|allele}$
**CN=4**	f(4)=f(dup)*f(dup)	**AAAA**	f(4)*(1-f(B))^4	$(RR_{CN\|allele})^2$
		**AAAB**	f(4)*4*f(B)*(1-f(B))^3	$RR_{allele\|CN}*(RR_{CN\|allele})^2$
		**AABB**	f(4)*6*(1-f(B))^2*f(B)^2	$(RR_{allele\|CN})^2*(RR_{CN\|allele})^2$
		**ABBB**	f(4)*4*(1-f(B))*f(B)^3	$(RR_{allele\|CN})^3*(RR_{CN\|allele})^2$
		**BBBB**	f(4)*f(B)^4	$(RR_{allele\|CN})^4*(RR_{CN\|allele})^2$
