# Supplementary material for: Advantage of Using Allele-Specific Copy Numbers When Testing for Association in Regions with Common Copy Number Variants
Source: PLoS One. 2013 Sep 10;8(9):e75350. doi: 10.1371/journal.pone.0075350 (PMC3769257; doi:10.1371/journal.pone.0075350)
Supplement: Table S6 — Expected allele-specific copy number states frequencies. The frequencies are displayed for each of the 36 frequency scenarios investigated. (PDF) [file pone.0075350.s012.pdf]

Table S6. **Expected allele-specific copy number states frequencies.** The frequencies are displayed for each of the 36 frequency scenarios investigated.

			CN=0	CN=1		CN=2			CN=3				CN=4				
**f(B)**	**CNV type**	**f(norm)**	**NULL**	**A**	**B**	**AA**	**AB**	**BB**	**AAA**	**AAB**	**ABB**	**BBB**	**AAAA**	**AAAB**	**AABB**	**ABBB**	**BBBB**
**0.05**	**Deletions**	**1/3**	0.4444	0.4222	0.0222	0.1003	0.0106	0.0003	-	-	-	-	-	-	-	-	-
		**0.5**	0.2500	0.4750	0.0250	0.2256	0.0238	0.0006	-	-	-	-	-	-	-	-	-
		**0.8**	0.0400	0.3040	0.0160	0.5776	0.0608	0.0016	-	-	-	-	-	-	-	-	-
	**Del & Dup**	**1/3**	0.1111	0.2111	0.0111	0.3008	0.0317	0.0008	0.1905	0.0301	0.0016	0.0000	0.0905	0.0191	0.0015	0.0001	0.0000
		**0.5**	0.0625	0.2375	0.0125	0.3384	0.0356	0.0009	0.2143	0.0338	0.0018	0.0000	0.0509	0.0107	0.0008	0.0000	0.0000
		**0.8**	0.0100	0.1520	0.0080	0.5957	0.0627	0.0017	0.1372	0.0217	0.0011	0.0000	0.0081	0.0017	0.0001	0.0000	0.0000
	**Duplications**	**1/3**	-	-	-	0.1003	0.0106	0.0003	0.3811	0.0602	0.0032	0.0001	0.3620	0.0762	0.0060	0.0002	0.0000
		**0.5**	-	-	-	0.2256	0.0238	0.0006	0.4287	0.0677	0.0036	0.0001	0.2036	0.0429	0.0034	0.0001	0.0000
		**0.8**	-	-	-	0.5776	0.0608	0.0016	0.2744	0.0433	0.0023	0.0000	0.0326	0.0069	0.0005	0.0000	0.0000
**0.2**	**Deletions**	**1/3**	0.4444	0.3556	0.0889	0.0711	0.0356	0.0044	-	-	-	-	-	-	-	-	-
		**0.5**	0.2500	0.4000	0.1000	0.1600	0.0800	0.0100	-	-	-	-	-	-	-	-	-
		**0.8**	0.0400	0.2560	0.0640	0.4096	0.2048	0.0256	-	-	-	-	-	-	-	-	-
	**Del & Dup**	**1/3**	0.1111	0.1778	0.0444	0.2133	0.1067	0.0133	0.1138	0.0853	0.0213	0.0018	0.0455	0.0455	0.0171	0.0028	0.0002
		**0.5**	0.0625	0.2000	0.0500	0.2400	0.1200	0.0150	0.1280	0.0960	0.0240	0.0020	0.0256	0.0256	0.0096	0.0016	0.0001
		**0.8**	0.0100	0.1280	0.0320	0.4224	0.2112	0.0264	0.0819	0.0614	0.0154	0.0013	0.0041	0.0041	0.0015	0.0003	0.0000
	**Duplications**	**1/3**	-	-	-	0.0711	0.0356	0.0044	0.2276	0.1707	0.0427	0.0036	0.1820	0.1820	0.0683	0.0114	0.0007
		**0.5**	-	-	-	0.1600	0.0800	0.0100	0.2560	0.1920	0.0480	0.0040	0.1024	0.1024	0.0384	0.0064	0.0004
		**0.8**	-	-	-	0.4096	0.2048	0.0256	0.1638	0.1229	0.0307	0.0026	0.0164	0.0164	0.0061	0.0010	0.0001
**0.35**	**Deletions**	**1/3**	0.4444	0.2889	0.1556	0.0469	0.0506	0.0136	-	-	-	-	-	-	-	-	-
		**0.5**	0.2500	0.3250	0.1750	0.1056	0.1138	0.0306	-	-	-	-	-	-	-	-	-
		**0.8**	0.0400	0.2080	0.1120	0.2704	0.2912	0.0784	-	-	-	-	-	-	-	-	-
	**Del & Dup**	**1/3**	0.1111	0.1444	0.0778	0.1408	0.1517	0.0408	0.0610	0.0986	0.0531	0.0095	0.0198	0.0427	0.0345	0.0124	0.0017
		**0.5**	0.0625	0.1625	0.0875	0.1584	0.1706	0.0459	0.0687	0.1109	0.0597	0.0107	0.0112	0.0240	0.0194	0.0070	0.0009
		**0.8**	0.0100	0.1040	0.0560	0.2789	0.3003	0.0809	0.0439	0.0710	0.0382	0.0069	0.0018	0.0038	0.0031	0.0011	0.0002
	**Duplications**	**1/3**	-	-	-	0.0469	0.0506	0.0136	0.1221	0.1972	0.1062	0.0191	0.0793	0.1709	0.1380	0.0495	0.0067
		**0.5**	-	-	-	0.1056	0.1138	0.0306	0.1373	0.2218	0.1194	0.0214	0.0446	0.0961	0.0776	0.0279	0.0038
		**0.8**	-	-	-	0.2704	0.2912	0.0784	0.0879	0.1420	0.0764	0.0137	0.0071	0.0154	0.0124	0.0045	0.0006
**0.5**	**Deletions**	**1/3**	0.4444	0.2222	0.2222	0.0278	0.0556	0.0278	-	-	-	-	-	-	-	-	-
		**0.5**	0.2500	0.2500	0.2500	0.0625	0.1250	0.0625	-	-	-	-	-	-	-	-	-
		**0.8**	0.0400	0.1600	0.1600	0.1600	0.3200	0.1600	-	-	-	-	-	-	-	-	-
	**Del & Dup**	**1/3**	0.1111	0.1111	0.1111	0.0833	0.1667	0.0833	0.0278	0.0833	0.0833	0.0278	0.0069	0.0278	0.0417	0.0278	0.0069
		**0.5**	0.0625	0.1250	0.1250	0.0938	0.1875	0.0938	0.0313	0.0938	0.0938	0.0313	0.0039	0.0156	0.0234	0.0156	0.0039
		**0.8**	0.0100	0.0800	0.0800	0.1650	0.3300	0.1650	0.0200	0.0600	0.0600	0.0200	0.0006	0.0025	0.0038	0.0025	0.0006
	**Duplications**	**1/3**	-	-	-	0.0278	0.0556	0.0278	0.0556	0.1667	0.1667	0.0556	0.0278	0.1111	0.1667	0.1111	0.0278
		**0.5**	-	-	-	0.0625	0.1250	0.0625	0.0625	0.1875	0.1875	0.0625	0.0156	0.0625	0.0938	0.0625	0.0156
		**0.8**	-	-	-	0.1600	0.3200	0.1600	0.0400	0.1200	0.1200	0.0400	0.0025	0.0100	0.0150	0.0100	0.0025
